# Beneficial Insect Attraction to Milkweeds (*Asclepias speciosa, Asclepias fascicularis*) in Washington State, USA

**DOI:** 10.3390/insects7030030

**Published:** 2016-06-29

**Authors:** David G. James, Lorraine Seymour, Gerry Lauby, Katie Buckley

**Affiliations:** Department of Entomology, Washington State University, Prosser, WA 99350, USA; lseymour@wsu.edu (L.S.); gerry@lauby.com (G.L.); katie.buckley@email.wsu.edu (K.B.)

**Keywords:** Milkweed, *Asclepias*, beneficial insects, conservation biological control, pollinators

## Abstract

Native plant and beneficial insect associations are relatively unstudied yet are important in native habitat restoration programs for improving and sustaining conservation biological control of arthropod pests in agricultural crops. Milkweeds (*Asclepias* spp.) are currently the focus of restoration programs in the USA aimed at reversing a decline in populations of the milkweed-dependent monarch butterfly (*Danaus plexippus*); however, little is known of the benefits of these plants to other beneficial insects. Beneficial insects (predators, parasitoids, pollinators) attracted to two milkweed species (*Asclepias speciosa*, *Asclepias fascicularis*) in central Washington State, WA, USA were identified and counted on transparent sticky traps attached to blooms over five seasons. Combining all categories of beneficial insects, means of 128 and 126 insects per trap were recorded for *A. speciosa* and *A. fascicularis*, respectively. Predatory and parasitic flies dominated trap catches for *A. speciosa* while parasitic wasps were the most commonly trapped beneficial insects on *A. fascicularis.* Bees were trapped commonly on both species, especially *A. speciosa* with native bees trapped in significantly greater numbers than honey bees. Beneficial insect attraction to *A. speciosa* and *A. fascicularis* was substantial. Therefore, these plants are ideal candidates for habitat restoration, intended to enhance conservation biological control, and for pollinator conservation. In central Washington, milkweed restoration programs for enhancement of *D. plexippus* populations should also provide benefits for pest suppression and pollinator conservation.

## 1. Introduction

Restoring native plants and habitats is increasingly seen as a critical part of enhancing and sustaining conservation biological control of insects and mites in agricultural crop pest management [1,2,3]. Native natural enemies co-evolved with native plants long before agriculture fragmented the landscape disrupting natural ecosystem services like the suppression of herbivores. Greater access to native plant resources should have a positive impact on the persistence and function of native beneficial insects like predators, parasitoids and pollinators in crop ecosystems. Beneficial insect and native plant associations are poorly studied in many regions; the identity of the most valuable native plants in terms of the beneficial insects they harbor and sustain is thus frequently unknown. Fortunately, this situation is improving with a number of recent studies showing the benefit of local native plants in enhancing attraction and sustenance of beneficial insects [4,5,6,7,8,9,10]. In Washington State, James et al. [11,12] reported substantial attraction of beneficial insects to flowering native buckwheats (*Eriogonum* spp.) and stinging nettles (*Urtica* *dioica* L.).

*Asclepias* L. is a genus in the Apocynaceae containing at least 76 species of perennial herbaceous plants known as “milkweeds” that occur throughout the United States into southern Canada [13]. Milkweeds are best known as the larval hosts of monarch butterflies (*Danaus plexippus* L.) and two other danaid species in North America [14]. An apparent decline in milkweed populations throughout the United States in recent years [15,16] has been blamed for a similar decline in populations of *D. plexippus* since the late 1990s [16]. Consequently, milkweed restoration efforts have been initiated by numerous private and public enterprises [17] as part of a federal campaign to help reverse the decline in monarch butterfly populations [18]. Organizations like the Xerces Society for Invertebrate Conservation have also promoted the cultivation and benefits of milkweeds for monarchs and pollinators generally [19]. The attraction of pollinators (bees, butterflies, moths, flies, beetles) to *Asclepias* spp. has been recognized for some time [20,21,22,23,24], but the value of *Asclepias* spp. to other beneficial insects like predators and parasitoids has not received the same attention. To date, the tachinid fly parasitoid of stink bugs, *Trichopoda pennipes* (F.), is the only natural enemy of a herbivore demonstrated to be attracted to a milkweed species (*Asclepias curassavica* L.) [25].

Here, we report the results of a field study on the attraction of predators, parasitoids and pollinators to the two endemic species of *Asclepias*, *A. speciosa* Torr. and *A. fascicularis* Decne occurring in an agriculturally intensive area of central Washington. Some horticultural industries in this region, for example wine grapes, have low inputs of pesticides and depend upon conservation biological control for much of their arthropod pest management [26,27]. Restoration of native flora and habitats is being pursued as a method of enhancing and sustaining the ecosystem services provided by natural enemies of pests as well aiding the conservation of threatened invertebrate fauna like butterflies [28].

## 2. Materials and Methods

### 2.1. Sites

**Figure 1:**
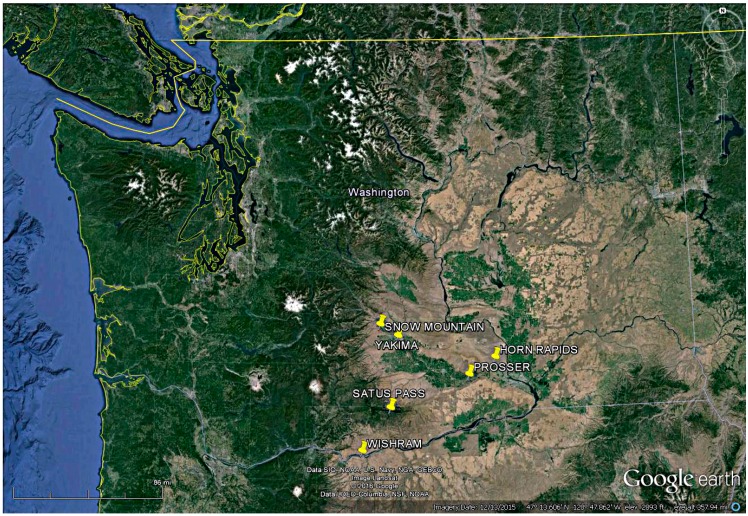
Washington State, WA, USA showing locations of *Asclepias speciosa* and *Asclepias fascicularis* sampled for beneficial insect attraction.

This study was conducted over four seasons (2010–2014) in central Washington by counting and identifying beneficial insects attracted to blooming showy milkweed (*Asclepias speciosa* Torr.) and narrow-leaved milkweed (*Asclepias fascicularis* Decne) using transparent sticky traps. Milkweed plants were located growing in riparian or natural areas at six locations (Wishram (45.40° N, 120.46° W), Satus pass (45.60° N, 120.38° W), Snow Mountain (46.39° N, 120.46° W), Yakima (46.32° N, 120.90° W), Prosser (46.14° N, 119.42° W), and Horn rapids (46.22° N, 119.26° W) (Figure 1). *Asclepias speciosa* was sampled at all sites except Wishram. *Asclepias fascicularis* was sampled at Wishram, Satus pass and Snow Mountain only. *Asclepias speciosa* and *A. fascicularis* are the only milkweed species present in Washington and the sampling sites used in this study for these plants are representative of the low-rainfall eastern areas of the state in which these milkweeds occur.

### 2.2. Traps

Transparent sticky traps (WindowBugCatcher, large 40.6 cm × 12.1 cm, Alpha Scents Inc., Portland, OR, USA) were used, avoiding trap color as a potential influence on insect attraction. Transparent sticky traps were used in earlier studies in Washington on predatory and parasitic insects attracted to native plants and were successful in trapping a wide diversity of these insects. Traps were attached to plants as soon as blooming commenced. At each site and on each occasion trapping was conducted, three traps were placed on three *Asclepias* plants. Plants with traps were at least 5 m from other plants/traps at each site and traps were attached to plants using flexible wires and positioned to provide a sticky surface immediately above or adjacent to the flowers. Traps were left in place for 12–14 days before removal and were replaced if blooming continued. In a few instances, follow-up trapping occurred on the same plants (when plant numbers were limited) but usually different plants were chosen. Traps collected from the field were transported to the laboratory and stored at −30 °C until examined under a stereomicroscope.

### 2.3. Trap Processing

All insects were identified to family or species and counted. The incidence and abundance of 34 species, genera or groups of winged beneficial insects were recorded (Table 1). Numbers of leafhopper (*Erythroneura* spp.) and lygus bug (*Lygus* spp.) pests were also recorded.

Beneficial insects were condensed into 10 categories: lacewings (Chrysopidae), ladybeetles (Coccinellidae), predatory true bugs (Miridae, Anthocoridae), predatory thrips (Aeolothripidae), predatory and parasitic flies (Syrphidae, Empididae, Dolichopodidae, Tachinidae), ichneumonid and braconid wasps (Ichneumonidae, Braconidae), *Anagrus* wasps (Mymaridae), other parasitic wasps (Pteromalidae, Eulophidae, Trichogrammatidae, Scelionidae) and bees (Apoidea). Bees were separated into honey bees (*Apis mellifera* L.) and native bees. Bumblebees and larger wasps such as yellow jackets and hornets were often able to extricate themselves from the sticky material.

### 2.4. Data Analysis

Trapping data were log (log x) transformed prior to analyses to improve normality of variances and then back-transformed for reporting. Student’s *t*-test was used for comparing two groups. Repeated-measures analysis of variance (ANOVA) with means separated using the Holm-Sidak method was used for comparing multiple groups (SigmaStat Version 3.0., SPSS Inc., San Jose, CA, USA).

## 3. Results

During 2010–2013, 121 traps were placed on flowering *A. speciosa* plants during the period May 16 to August 25. Fifteen traps were used on flowering *A. fascicularis* during June 24–10 and July 2012–2014 (Table 2). The scarcity of *A. fascicularis* prevented trapping on a greater number of plants.

Beneficial insects dominated trap catches throughout the study. Very few pest insects were encountered. The only pests recorded were lygus bugs (*Lygus* spp.), grape leafhoppers (*Erythroneura* spp.) and western flower thrips (*Frankliniella occidentalis* (Pergande)). Traps on *A. speciosa* caught 0.2 and 0.05 individuals per trap of *Lygus* bugs and leafhoppers, respectively, during trapping from 2010–2013. Numbers of *F. occidentalis* were not recorded but were usually less than 10 per trap.

Combining all categories of beneficial insects and analyzed over all trapping years for each *Asclepias* species, the mean number per trap for *A. speciosa* was 121.8 ± 7.6 and for *A. fascicularis,* 125.8 ± 51.5. Comparing the two species during the years both were trapped (2012, 2013) showed no significant difference in numbers of beneficial insects trapped on *A. speciosa* (97.6 ± 9.2) and *A. fascicularis* (105.6 ± 7.6) (*t* = −1.299, df = 29, *p* = 0.204).

**Figure 2:**
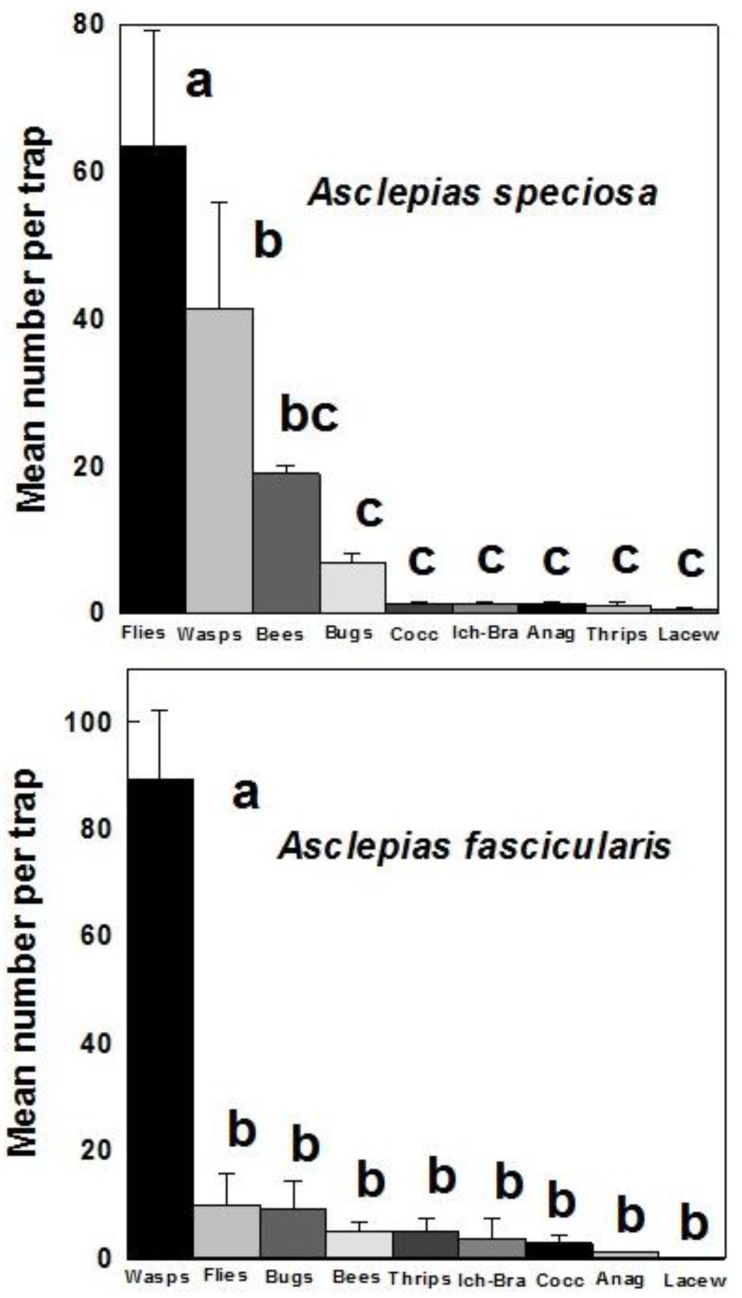
Mean (±SE) number per trap of different categories of beneficial insects trapped on flowering *A. speciosa* and *A. fascicularis* during 2013 (*speciosa*) or 2012–2014 (*fascicularis*). Bars denoted by different letters are significantly different (*p* < 0.001).

Significantly greater numbers of predatory and parasitic flies were attracted to *A. speciosa* than any other beneficial insect group (mean: 63.7/trap) (*F* = 14.721; df = 8, 278; *p* < 0.001) (Figure 2).

Parasitic wasps (41.4), bees (19.0) and predatory bugs (7.0) were the next most commonly attracted beneficials with relatively small numbers of coccinellids (1.4), ichneumonids/braconids (1.4), mymarids (1.4), predatory thrips (1.1) and lacewings (0.6) trapped (Figure 2). Parasitic wasps were the most commonly trapped beneficial insects on *A. fascicularis* (mean: 89.3/trap) (*F* = 49.631; df = 8, 112; *p* < 0.001) (Figure 2). Predatory and parasitic flies was the next most attracted group (10.1), followed by predatory bugs (9.2), bees (5.2), predatory thrips (5.1), ichneumonids/braconids (3.7). coccinellids (2.9), mymarids (1.1) and lacewings (0.2) (Figure 2).

**Figure 3:**
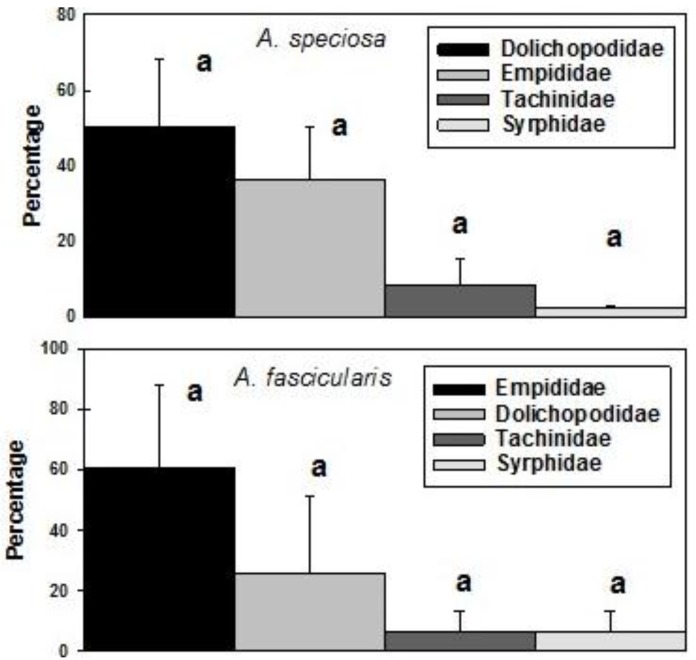
Mean (±SE) percentage per season of families comprising the predatory and parasitic fly category trapped on *A. speciosa* and *A. fascicularis* during 2010–2013 (*speciosa*) and 2012–2014 (*fascicularis*). Bars denoted by the same letter are not significantly different (*p* = 0.05).

The dominant beneficial fly families trapped on both *Asclepias* spp. were Dolichopodidae (long-legged flies) and Empididae (dagger flies) accounting for 19%–50% of flies trapped. Tachinids accounted for 3.0%–7.0% and syrphids 0.5%–2.0% (Figure 3). A total of 3398 flies were found on *A.* *speciosa* traps and 186 on *A. fascicularis* traps. Substantial temporal variability in the numbers of attracted dolichopodids, empidids and tachinids masked any statistical differences between the fly families (*F* = 2.752, df = 3, 9, *p* = 0.104 (*speciosa*); *F* = 1.607, df = 4, 12, *p* = 1.607 (*fascicularis*)) (Figure 3).

**Figure 4:**
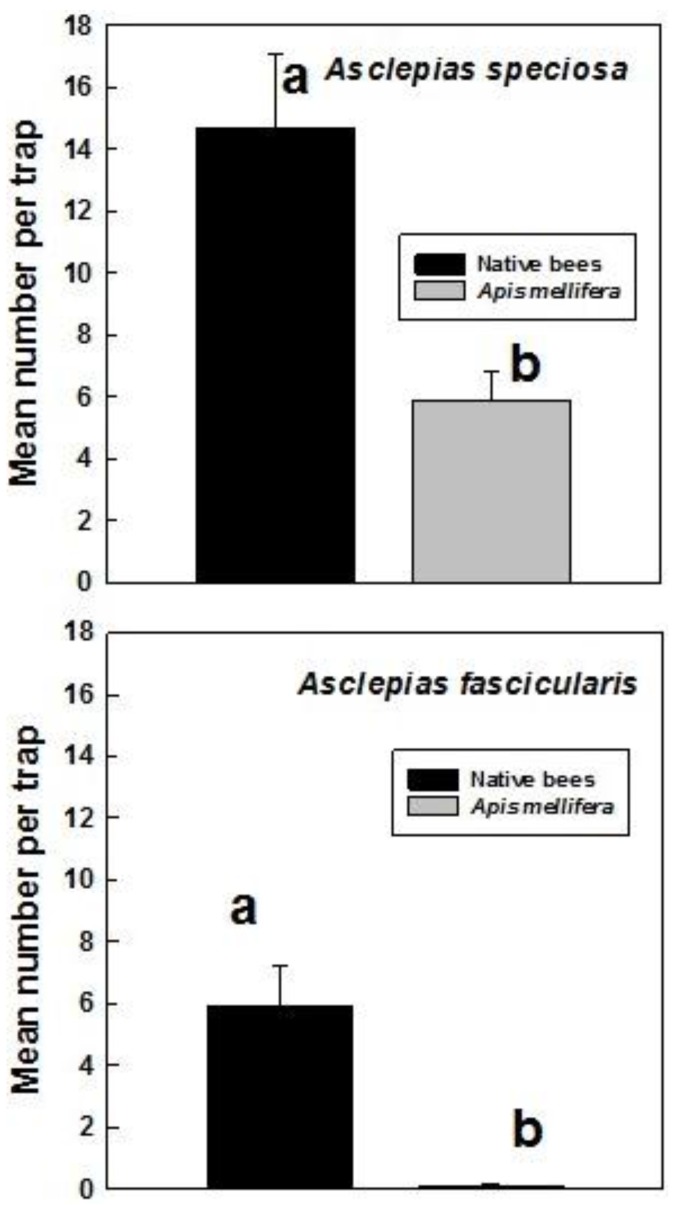
Mean (±SE) number of native bees and honey bees (*Apis mellifera*) trapped on flowering *A. speciosa* and *A. fascicularis* during 2010–2013 (*speciosa*) and 2012–2014 (*fascicularis*). Bars denoted by different letter are significantly different (*p* < 0.001).

The relative numbers of honey bees (*Apis mellifera* L.) and native bees trapped on the two *Asclepias* spp. was assessed in 2013 (*speciosa*) and 2012–2013 (*fascicularis*). A significantly greater numbers of native bees (mean 14.7/trap) than honey bees (5.9/trap) were trapped on *A. speciosa* (*t* = 3.403, df = 54, *p* = 0.001). Similarly, greater numbers of native bees (5.9/trap) than honey bees (0.1/trap) were trapped on *A. fascicularis* (*t* = 4.401, df = 26, *p* = 0.001) (Figure 4).

**Figure 5:**
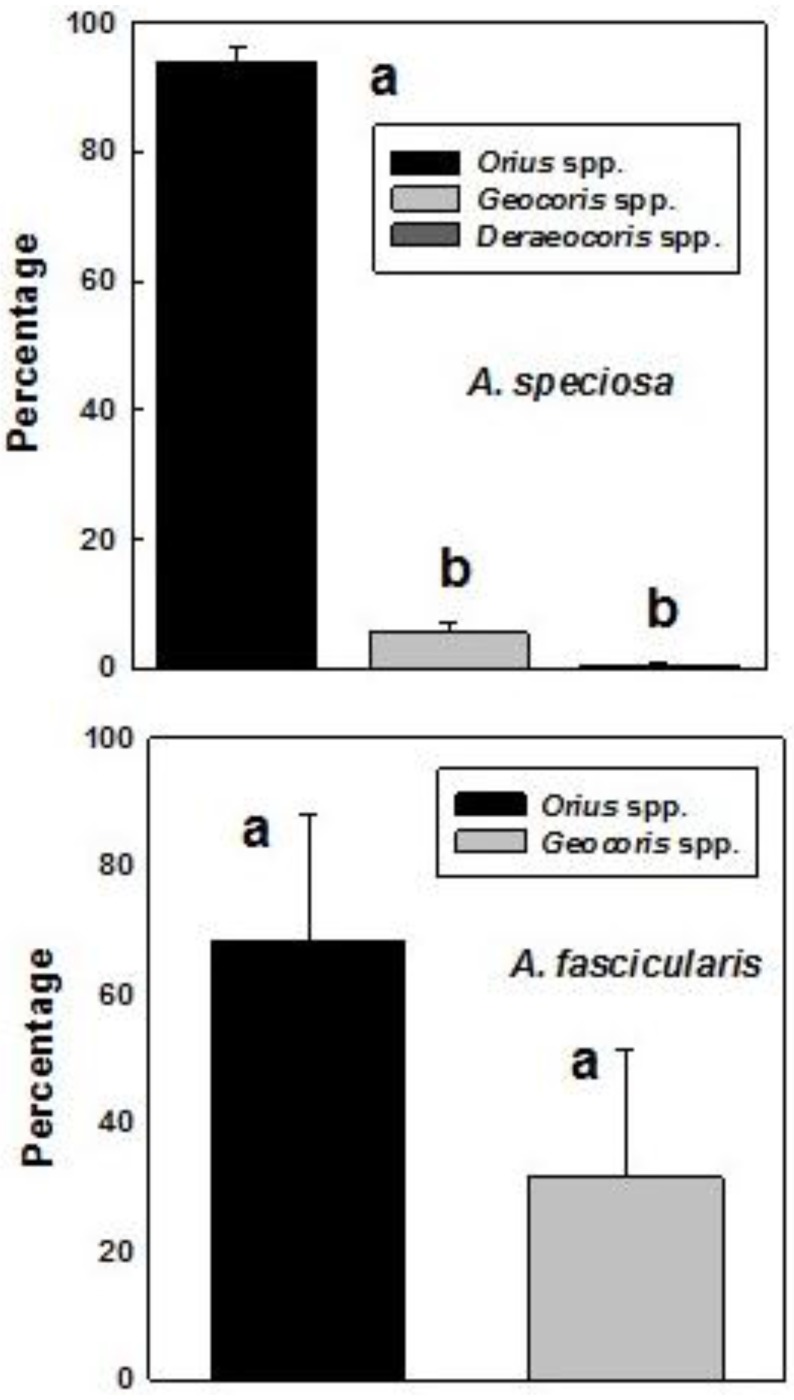
Mean (±SE) percentage per season of predatory bug genera (*Orius* spp., *Geocoris* spp., *Deraeocoris* spp.) trapped on flowering *Asclepias speciosa* and *Asclepias fascicularis* during 2010–2013 (*Asclepias)* and 2012–2014 (*fascicularis*). Bars denoted by different letters are significantly different (*p* < 0.001).

Combining data from all years, aphidophagous coccinellids (107) significantly outnumbered acariphagous coccinellids (35) trapped on *A. speciosa* (*t* = 6.75, df = 4, *p* = 0.0013) but were comparable in numbers (53, 55) on *A. fascicularis* (*t* = 0.399, df = 4, *p* = 0.728). Minute pirate bugs (*Orius* spp.) were the dominant predatory bugs trapped on both *Asclepias* spp., significantly so on *A. speciosa* (*F* = 659.4, df = 3, 11, *p* < 0.001) (Figure 5). Combining data from all years, 809 *Orius* spp. were trapped on *A. speciosa* and 158 on *A. fascicularis*.

## 4. Discussion

Milkweeds are currently receiving much attention and interest in North America because of their role as the sole host plant for immature stages of the monarch butterfly (*Danaus plexippus*) which has suffered a substantial population decline during the past two decades [29]. Many scientists consider a corresponding decline in milkweed populations to be the major factor responsible for reduced populations of *D. plexippus* [16]. Restoration of milkweeds throughout the United States is an ongoing component of programs being implemented by federal and state agencies to reverse the decline in monarch butterfly populations, as part of an initiative announced by the federal government in May 2015 to promote the health of pollinators [18]. Private citizens are also an enthusiastic part of this milkweed restoration program [30]. Other benefits of milkweeds beyond being the host plant for *D. plexippus* and being attractive to pollinators [22] have not been well explored. This study provides data on the attraction of a variety of insect predators and parasitoids to the two milkweed species present in Washington State.

Combining data for all categories and all years, *A. speciosa* and *A. fascicularis* attracted a similar number of beneficial insects (means 121.8, 125.8) per trap. Of 100 flowering plant species evaluated by our laboratory for beneficial insect attraction in central Washington, *A. speciosa* and *A. fascicularis* are ranked in the top 20. Ten species of flowering buckwheats (*Eriogonum* spp.) attracted means of 48.5–167.7 beneficial insects per trap and were considered to have potential in habitat-restoration strategies for improving biological control in Washington viticulture [11]. Sticky-trapping (same protocols) of *Medicago sativa* L. (alfalfa), considered highly attractive to beneficial insects [31] in July 2011 yielded a mean of 46.3 beneficial insects per trap. *Asclepias speciosa* and *A. fascicularis* therefore appear to be at the higher end of the beneficial insect attraction scale and should have value in habitat-restoration programs aimed at improving crop pest management.

Carnivorous flies were the dominant beneficial insects attracted to *A*. *speciosa* (>60/trap). Most of these insects were dolichopodids (long-legged flies) and empidids (dagger flies). Empidids both as adults and larvae are predatory on a wide range of arthropods from aphids to mosquito larvae [32] as are dolichopodids [33]. Tachinid flies were occasionally trapped in large numbers (50–60/trap). Tachinid flies, *Trichopoda pennipes*, were attracted to tropical milkweed (*Asclepias currassavica*) in Georgia, GA, USA [25] and this family of flies may be widely attracted to *Asclepias* spp. Tachinid fly attraction to milkweeds may be a factor in the importance of these parasitoids in the regulation of *D. plexippus* larval populations [34]. In contrast to the two *Asclepias* spp., carnivorous flies were not greatly attracted to *Eriogonum* spp. or stinging nettles (*Urtica dioica* L.) [11,12].

Predatory and parasitic flies did not appear to be strongly attracted to *A. fascicularis* (mean 10.1/trap) with parasitic wasps (Pteromalidae, Eulophidae, Trichogrammatidae, Scelionidae) dominating the trap catches for this species (mean 89.3/trap). Parasitic wasps were the second most numerous group of beneficial insects attracted to *A. speciosa* (mean 41.4/trap).

Bees were strongly attracted to *A. speciosa* (mean 20.6/trap) comparable to *Eriogonum niveum* Douglas ex Benth., a buckwheat species that attracted most bees in a central Washington study [11]. In contrast, *A. fascicularis* attracted few bees (mean 6.0/trap). Native bees accounted for 71% of the bees attracted to *A. speciosa* and 98% of the bees attracted to *A. fascicularis.* In contrast, nearly six times as many honeybees as native bees were recorded visiting blooms of *Asclepias incarnata* L. in Michigan, USA [35]. Honeybees also dominated bee pollinator visits to *Asclepias syriaca* L. in Illinois [21]. Studies of pollinators visiting four *Asclepias* species in Indiana and Arizona showed that the relative frequency of visits by honeybees and native bees (primarily bumblebees) varied but overall was balanced [22,24]. Bumblebees were not recorded in our study so it is likely that we underestimated native bee visitation to *A. speciosa* and *A. fascicularis.* Most of our trapping sites were in agricultural areas with high numbers of honeybees.

Predatory bugs, primarily *Orius* spp. (Anthocoridae), were trapped in good numbers on *A. speciosa* and *A. fascicularis* (means 7.0, 9.2/trap). They are also strongly attracted to flowering and non-flowering stinging nettles in central Washington [12] and some species of *Eriogonum* [11]. *Orius* spp. comprised 94% of the predatory bugs we trapped in this study and accounted for >95% of bugs in James et al. [12]. *Orius tristicolor* (White) was recorded on 64 plant species including *A. speciosa* in a central Washington study [36].

Other groups of beneficial insects were trapped at levels <2.0/trap. The number of ladybeetles trapped (mean 1.4/trap) was similar to numbers trapped on most *Eriogonum* spp. [11] and stinging nettles [12]. Milkweeds are often attacked by oleander aphids (*Aphis nerii* Boyer de Fonscolombe) but they were absent in this study. When present, they will likely increase attraction of aphidophagous ladybeetles as well as other aphid predators. Milkweeds rarely support mite populations and only 58 mite-feeding ladybeetles were trapped during our five-year study.

## 5. Conclusions

This study has shown that the two milkweed species occurring in central Washington, *A. speciosa* and *A. fascicularis,* attract a range of predators, parasitoids and pollinators during their blooming period from May−August. This is the first detailed evaluation of *Asclepias* spp. as pest natural enemy attractants and hopefully will encourage similar investigations on other milkweed species. Provision of a milkweed (*Asclepias currassavica*) insectary habitat in peanut-cotton farmscapes in Georgia, GA, USA increased tachinid fly parasitism of pest stink bugs, while aiding monarch butterfly and pollinator conservation [25]. Clearly, there is potential for *A. speciosa* and *A. fascicularis* to provide a similar role enhancing pest management in central Washington agriculture. The beneficial insects most attracted to *A. speciosa* and *A. fascicularis* (parasitic wasps, carnivorous flies, predatory bugs) play a significant role in suppressing a range of pest insects (e.g., aphids, leafhoppers, mealybugs, caterpillars, thrips) affecting a variety of crops (e.g., grapes, apples, hops, berries, cherries) in central Washington. Planting *A. speciosa* and/or *A. fascicularis* in non-cropped locations (e.g., corners of crop circles, ditches, rocky sites) near to crops should provide benefits to biological control and integrated pest-management programs. Benefits will likely vary according to the pests and natural enemies involved, but the use of milkweeds as plants that enhance and sustain biological pest management clearly deserves evaluation in specific locations and crops. *Asclepias speciosa* also appears to be a significant resource for native bees which are receiving increased attention as important pollinators for some crops [37]. *Asclepias speciosa* and *A. fascicularis* are two of the major milkweed species used as larval hosts of monarch butterflies in the western U.S. Significantly, conservation and proliferation of *A. speciosa* and *A. fascicularis* are critical to increasing populations of *D. plexippus* in the west [38]. The information presented here on the value of these milkweed species in attracting beneficial insects provides an additional and supporting reason for cultivating these plants in farmscapes or landscapes generally.

## Figures and Tables

**Table 1:** Categories of beneficial insects identified and recorded in this study, along with species, genera and families within each category.

Beneficial Insect Categories	Species, Genera or Family Included
Neuroptera (Lacewings)	*Chrysoperla plorabunda* (Fitch) *Chrysopa nigricornis* Burmeister *Chrysopa coloradensis* Banks *Chrysopa oculata* Say
Coccinellidae (Ladybeetles)	*Harmonia axyridis* (Pallas) *Coccinella septempunctata* L. *Coccinella transversogutatta* Mulsant *Hippodamia convergens* (Guerin-Meneville) *Stethorus picipes* Casey *Stethorus punctillum* (Weise)
Heteroptera (Predatory bugs)	*Deraeocoris brevis* (Uhler) *Geocoris pallens* Stal *Orius* spp.
Aeolothripidae (Predatory thrips)	*Franklinothrips* spp. *Aeolothrips* spp.
Diptera (Predatory and parasitic flies)	Empididae Syrphidae Dolichopodidae Tachinidae
Icheumonidae and Braconidae (Ichneumonid and braconid wasps)	
Mymaridae (Fairy flies)	*Anagrus* spp.
Other parasitic wasps	Pteromalidae, Eulophidae, Trichogrammatidae, Scelionidae
Apoidea (Bees)	*Apis mellifera* L., Andrenidae, Halictidae, Megachilidae, Apidae, Colletidae

**Table 2:** Number of traps used and trapping period for *A. speciosa* and *A. fascicularis* during 2010–2014 in central Washington.

Year	*A. speciosa*		*A. fascicularis*	
	No. of Traps	Trapping Period	No. of Traps	Trapping Period
2010	12	28 June–19 July	0	–
2011	30	19 July–25 August	0	–
2012	51	16 May–23 July	6	26 June–10 July
2013	28	11 June–24 July	6	27 June–10 July
2014	0	–	3	24 June–8 July
All years	121	16 May–25 August	15	24 June–10 July
